# Structured medical electives: a concept whose time has come?

**DOI:** 10.1186/s12992-019-0526-2

**Published:** 2019-12-03

**Authors:** Chris Willott, Eva Khair, Roger Worthington, Katy Daniels, A. Mark Clarfield

**Affiliations:** 1 King’s Centre for Global Health and Health Partnerships, King’s College London, London, UK; 2 Independent Researcher, London, UK; 3 University of Dundee Medical School, Dundee, UK; 4 Medical School for International Health, Ben-Gurion University of the Negev, Beer-sheva, Israel

**Keywords:** Elective, Structured elective, Global health, Ethics, Curriculum

## Abstract

**Background:**

Most international electives in which medical students from high-income countries travel abroad are largely unstructured, and can lead to problematic outcomes for students as well as sending and receiving institutions. We analyse the problems of unstructured medical electives and describe the benefits of an elective experience that includes more organisation and oversight from the sending medical school.

**Results:**

A number of structured elective programmes have been developed, including those at the Medical School for International Health, Israel and the University of Dundee, United Kingdom. These programmes provide significant pre-departure training in global health and the ethical dimensions of electives, support and monitoring during the elective, and post-elective debrief. Crucially, the programmes themselves are developed on the basis of long-term engagement between institutions, and have an element of reciprocity.

We further identify two major problems in current medical electives: the different ethical contexts in which electives take place, and the problem of ‘voluntourism’, in which the primary beneficiary of the activity is the medical student, rather than the receiving institution or health system. These two issues should be seen in the light of unequal relations between sending and receiving institutions, which largely mirror unequal relations between the Global North and South.

**Conclusion:**

We argue that more structured elective programmes could form a useful corrective to some of the problems identified with medical electives. We recommend that medical schools in countries such as the UK strongly consider developing these types of programmes, and if this is not possible, they should seek to further develop their pre-departure training curricula.

## Background

International medical electives (IME) are a well-established part of curricula at most medical schools in high-income countries (HIC) such as the UK, Australia and Canada [1–3]. Many schools allow or in some cases encourage their students to avail themselves of these experiences. For example, of the British students undertaking an IME, approximately 40% do so in a low-income country (LIC) (Miranda et al., 2005); in Australia this can be as high as 59% [2].

A 2002 UK survey estimated that 40% of undergraduate medical electives were being spent in LICs, representing a collective 350 student-years of elective time spent *annually* by UK medical students alone [4]. These authors further highlight evidence that elective attachments are largely “one-way”, tilting “very much in favour of students visiting from wealthy countries” and that such “medical tourism” offered only sporadic benefits to hosts in developing countries.

Despite efforts made to address these concerns, at many medical schools, rhetoric and action about electives can still be a long way apart: even if sending institutions have concerns about the impact of their students’ activities, mandate that students pre-prepare objectives and receive guidance about what is expected of them during elective, effective monitoring of students’ activities on the ground is nearly impossible. This raises an issue for the future of such medical electives, because there is an inherent tension: students frequently want to be able to decide for themselves where they go and how they spend their electives, but this may not be what is best for hosting institutions, nor for global health more generally. Sending medical schools are also unlikely to possess the resources to develop structured elective programmes for students.

For many, electives are about discovering a new culture, health system and disease burden, but for others it can comprise a ‘treat’ at the end of a difficult and exacting degree. Some students may choose to spend their time on the beach or in other holiday destinations, rather than working clinically. Conversely, in terms of equity and ethics, we would argue that more structured electives, with greater peer support, monitoring and pre-departure training, could engender better quality elective experiences that are more sustainable for all.

To address relevant issues surrounding IMEs, a group of academics, electives organisers and students met in February 2019 under the auspices of the Royal Society of Medicine (RSM) in London, to discuss how electives can better serve everyone involved in them – students and medical schools in sending countries, and medical schools, electives organisers and mentors, fellow students and the patient population in receiving countries. We should note here that the majority of our discussion focused on the sending of medical (and nursing and other allied health professional) students from HICs to other places, whether High-Income or Low and Middle Income (LMIC). With the exception of a discussion of the electives programme at the Ben-Gurion University of the Negev in Israel, which described the experiences of a medical school whose whole curriculum is suffused with Global Health instruction, our deliberations focused primarily around UK medical schools. That being said, the issues addressed would be relevant to any HIC medical school that sends or allows its students to do an “away” elective.

The main conclusions arising from these discussions focus on the content and value of structured elective programmes for students and the importance of embedding ethical principles into all aspects of elective design and practice.

### Structured elective programmes

Fully-structured programmes, in which students receive significant support from their medical schools in the form of pre-departure training and support during their electives, may be a good way to reduce negative implications of electives for receiving countries and also provide students with a programme in which they are able to learn about global health in a more structured way. Here we outline two programmes that seek to provide, for want of a better term, ‘ethical electives’ for their students.

### Ben-Gurion University of the Negev, Beer-sheva, Israel

The Medical School for International Health (MSIH) is a four-year North American style institution based at the Ben-Gurion University of the Negev [5, 6]. Opened more than two decades ago, this institution offers a medical degree to students with a first degree (BA or BSc in any subject). Global Health (GH) themes are embedded throughout the curriculum, including the relevant cross-cultural and ethical challenges that may exercise them during their IME. As a capstone experience, in the fourth and final year all students are assigned for 8 weeks to one of nine partner sites in seven LMICs: Ghana, Ethiopia, India (three sites), Nepal, Sri Lanka, Mexico and Peru. This rotation is mandatory and MSIH manages to match most students’ wishes, with 95% getting their first or second choice site.

While this school exists within a non-profit university, tuition covers the total cost of these rotations apart from room and board. MSIH has a long-standing relationship with these LMIC institutions and pays students’ flight expenses as well as tuition directly to the institutions. In an effort to maintain two-way learning, MSIH also has a reciprocal arrangement whereby scholarship students (fully paid for by MSIH) from both Ghana and Ethiopia travel to Israel for 12 weeks where they choose from a palette of clinical rotations at MSIH’s main teaching hospital. Students going in both directions interact at their respective teaching sites. This provides an excellent opportunity for both sides to learn from each other from being immersed in the other’s clinical milieu. Over the past decade MSIH has also held two academic conferences dealing with the clinical and ethical aspects of the exchange programme, where representatives from all of its nine LMIC sites are invited to participate. All costs of their flights and stay in Israel are covered by MSIH.

For the students travelling to LMICs, the overarching goal is for them to apply what they have learned in the first 3 years of their GH studies and to confront the challenges of caring for patients in severely resource-challenged sites – both in the hospital and the community.

Close attention is paid to a formal pre-rotation orientation in all relevant domains, especially but not exclusively related to health and safety. In addition to these practical issues, in their third year of study students receive comprehensive instruction in the relevant cross-cultural aspects of clinical care via a two-day intensive simulation programme using actors [5, 6]. Issues such as how to cope with different health belief models, ‘choosing wisely’ in low resource settings and how to practice cultural humility are all specifically addressed. Faculty observe students in a small group setting and constructively critique their performance with special emphasis on relevant situations that they likely will face when abroad.

In addition, in order to maximise student safety as well as to keep track of their clinical and learning experiences, students are required to write a twice-weekly blog, which is read by assigned faculty members and open only to this class. The faculty supports a frank expression of views and concerns and students are encouraged to share both clinical and cultural experiences, including ethical and/or clinical dilemmas they have encountered during their rotation.

Should any learner feel the need to connect individually with faculty for any reason, they may do so directly, outside of the blog. While the purpose of this blog is primarily educational, there have been situations when faculty have picked up issues relating to a student’s physical or mental health. These have been dealt with either via email or telephone. If necessary, MSIH staff connect with our on-site partners abroad.

Over 20 years, MSIH has offered approximately 1500 student-months of GH learning experience. There has been no systematic evaluation of the programme, but feedback from students garnered each year from semi-formal debriefing sessions has been almost universally positive, specifically with respect to the medical education goals set as well as regarding student exposure to the “hidden” curriculum that all learners face when exposed to difficult conditions. Feedback received in the semi-formal debriefings held on their return shows that almost all students report having learned a great deal and a large minority have described these experiences as ‘transformative’ [7]. From January 2020 onwards MSIH intends to formally collate and summarise the results of these debriefing sessions.

While students in both directions pay nothing for their clinical experiences, such an exchange programme has opportunity costs. While MSIH reimburses the ‘away’ schools, faculty and administrative staff at both sites (home and away) must invest significant effort to ensure that things work according to plan. Furthermore, MSIH faculty make regular visits to the away sites in order to monitor the programmes.

While minor challenges are frequently encountered, MSIH has been providing this rotation for just over two decades without experiencing any major problems. Despite this, the programme is a work in progress and requires constant monitoring with subsequent adjustment.

### Dundee University medical school, Scotland, UK

Dundee Medical School runs a five-year undergraduate medicine degree. An elective is a core part of the curriculum and takes place in the fifth year. Students have an eight-week elective period, during which they complete a minimum of 6 weeks clinical attachment. Electives are organised by the students themselves with support from faculty and are usually self-funded, as is typical for UK medical schools, though some bursaries are available. Destination is largely only limited by Foreign and Commonwealth Office (FCO) guidance. Irrespective of destination, all students are required to complete four online preparation modules (Table 1) to promote positive attitudes and cultural humility whilst equipping students for their elective. Students must also submit a proposal with the rationale for their chosen elective, five objectives (two of which have to have explicit global health themes) and a risk assessment before their elective is approved. Elective assessment takes the form of a reflective report discussing the five objectives chosen and a supervisor’s assessment form on which written feedback is given.

**Table 1:** Dundee medical school online preparation modules

Module Title	Themes of module content
Planning your elective	Aims of electives; factors influencing destination and specialty choices; concept of medical tourism; attitude to their elective; potential language, culture and ethical issues
Knowing your environment	Introduces global health issues, students research global health issues at their elective destination
Thinking about risk	Explores risks under seven themes: communication, personal health, clinical risks, accommodation, travel and leisure, people and culture and regional factors.
Elective ethics	Using case examples explores the impact of language barriers; working within competency; impact of limited resources; decision making in different cultures and consent

Dundee Medical School first piloted a ‘Responsible Elective’ (RE) model in 2006 in Malawi, aided by a Scottish government grant, using a long-term partnership model. From the outset the aim was to promote ethical electives, recognising that these experiences, particularly in LICs, can consume already limited resources at host hospitals with limited benefits for them. As such, core values for the Responsible Elective programme were developed within five categories: ethical, educational, evaluated, environmental and social. (For more information on these core values, see https://blogs.cmdn.dundee.ac.uk/responsible-electives/our-ethos/).

‘Responsible Electives’ sought to achieve these core values through formal partnerships with health institutions in developing countries, linking UK medical students with existing overseas partner sites, providing pre-departure training and encouraging students to fundraise for the benefit of their hosts. The aim was to optimise student learning, including developing a strong sense of global citizenship and promoting a more considered and fairer ‘trade’ in electives, where host sites benefit.

In the first 6 years, Dundee medical students who opted to participate were placed on extended electives (4 months) in Malawi and the funding also supported two students each year from College of Medicine, Malawi, to undertake a six-week elective in Dundee.

The Responsible Elective programme has evolved with time, based on feedback from students and sub-Saharan elective hosts [8]. Two partner sites exist, one in Malawi and one in Zambia, and a staff visit in September 2015 resulted in a renewed interest to continue and develop the RE programme further. A Memorandum of Understanding was agreed with each host site, outlining expectations of both parties, guaranteeing to provide six student places across the year and reduced costs for students participating by approximately £250 per student. In return, the School of Medicine makes an annual payment of £2000 to each hospital and students are expected to fundraise for their host hospital, each being given a £1000 target, which many students exceed. Students are also given an option of an extended elective (10 weeks clinical rather than the standard six) because hosts suggested that this model was of more benefit to them.

The relationship with these elective hosts is fostered by regular direct communication between partner hospital staff and Dundee medical school, a practice that does not take place in ‘standard’ electives. This allows areas of good practice to be highlighted and any challenges to be identified and addressed promptly. A member of Dundee medical staff also spends approximately 6 weeks a year working at one of the partner sites, partly linked to International health BMScs as students undertake research projects for them. Although this is not part of the elective programme, it highlights the broader relationship that has been developed.

From 2015 to 19, 47 students participated in RE, with 26 completing an extended elective. Feedback from students and hosts has remained positive over this time. Participants’ feedback to staff and peers is that REs provide an improved educational experience when compared to similar electives. It is believed that this feedback and communication among peers is the driving factor for increasing student demand as well as increased awareness of ethical issues posed by electives.

A few minor challenges have occurred, the main ones due to miscommunication at the changeover of hospital management, however regular communication have been addressed promptly. Adverse events (such as blood borne virus exposure, theft) are infrequent. From 2015 to 18, nearly £37,000 has been raised by students for host hospitals. Importantly, the hospitals decide how this money is used, recognising that they are best placed to know their needs, which has included building renovations, purchasing equipment and supplies and providing funding for local staff training.

The recent launch of the Scottish Graduate Entry Medicine course (https://www.st-andrews.ac.uk/subjects/medicine/scotgem-mbchb/) has created an opportunity to develop and expand the RE programme due to the interest from the majority of first year students, alongside the increasing demand for more ethical electives from Dundee medical students. As such, the hope is to use newly available resources to introduce additional host sites, increasing capacity, and explore further how reciprocity can be achieved with hosts, bearing in mind the ethical challenges electives can pose. These additional resources will also allow for structured evaluation of the RE programme which includes an upcoming meeting with senior staff from one partner site during a visit to Scotland to discuss the current programme and opportunities for development.

### Ethical challenges

An important theme that developed during the course of the day focused on ethical considerations and how medical schools and students attempt to deal with them. One key challenge for the sending institution lies in guiding students on how to behave in a context that is often very different from their own. We examined three elements: clinical, professional and social. For UK medical schools, the General Medical Council states that their guidelines should be adhered to at all times whilst on elective [9], but however necessary, these instructions may not be sufficient for students going on certain types of international electives.

Firstly, ethics may be conceptualised differently in countries where students go on elective, and core concepts in ethics, such as respect for patient autonomy, could have a different meaning, where for example family plays a dominant role and patients are not told their diagnosis [10, 11]. Furthermore, contact time with patients is sometimes so limited that there is no opportunity for discussion or any meaningful choice other than to forgo treatment, as typically happens in public hospitals in India [12]. Notions of informed consent currently in place in most HICs can lack contextual validity in settings such as these, and students need to be prepared and know how to best respond.

Similarly, professionalism may work differently from in the UK, and culturally-rooted paternalistic behaviour patterns are still common in many LMICs [13]. Such behaviours are contrary to standards set by the GMC, which reflects British cultural norms [14], and in places where paternalism is still widely practised, students need to be sensitized to possible cultural misunderstandings. Female students in particular also need to be careful in case they receive any inappropriate attention from local male colleagues, especially in the case of senior clinicians [15].

Other ethical problems relate to social conditions and the impact on society of student doctors giving clinical assistance for short periods and then leaving. While we discuss this issue below in more detail, it is worth noting that where patients might have had no access to medical professionals for long periods of time [16–18], visiting medical students can be seen as being no different from qualified medical professionals, which is hazardous in both directions in terms of expectations. An additional hazard could lie in students engaging in local healthcare politics, which with all its hidden complexities, would be very ill-advised, especially if corruption is an entrenched problem [19].

It also behoves us as well as our students to recall that it was not too long ago that in HICs, many practices observed in LICs which we may now consider to be wrong or misguided were also common in HICs. Furthermore, in situations such as for example, sexual harassment, HICs still have a way to go. Simply put, when one travels and works abroad, especially in the field of GH, one should practice ‘cultural humility’ whenever possible.

The notion of cultural humility, defined as ‘having an interpersonal stance that is other-oriented rather than self-focused, characterized by respect and lack of superiority towards an individual’s cultural background and experience’ [20], is not easy to teach or to engender in students who are about to travel abroad for their electives. Structured electives are beneficial for cultural humility because, first, students have the opportunity to actively consider different scenarios they may face and how to deal with them sensitively and ethically; and second, because structured programmes, with strong ties between institutions, mean that any student who behaves in a way that is inappropriate will be flagged to their home institution. This is much more difficult in unstructured electives.

In programmes such as those described here, the overarching moral question may be borrowed from the legal profession: *‘cui bono?’* namely ‘*for whose benefit is a programme being run*?’. Medical electives should be valuable learning experiences for students who choose to make the most of the available opportunities [21], but if, for instance, there is no continuity of care for patients after students return home, the local impact could be negative. Medical schools have an ethical responsibility to be mindful of the impact that electives may have on local communities and to prepare students accordingly [22]. Leaving students to ‘do their own thing’ could even be seen as derogation of the duty of care towards students, especially if there is limited clinical supervision or provision for students’ physical safety and security [23].

In short, there are ethical and professional arguments for ensuring that a) medical schools assume responsibility for the actions of their students whilst on elective and train their own staff accordingly; b) students are properly briefed pre-departure and de-briefed on return, and c) faculty connections are maintained with local service providers after students leave. Initiatives such as those described at Ben Gurion and Dundee Universities that limit choices open to students could be seen as good models for medical schools to adopt in the UK and elsewhere.

### Medical electives as ‘voluntourism’

A related issue is the degree to which electives can be conceptualised as a form of ‘voluntourism’, in which the students benefit but the medical school and the broader health systems in receiving countries lose out. Definitions of volunteer tourism as ‘utilizing discretionary time and income to go out of the regular sphere of activity to assist others in need’ [24] and further conceptualisation of it as a sort of ‘moral tourism’, whose aim ‘is to assist conservation and community well-being goals in the Global South’ [25], show clear commonality with an HIC medical elective experience, particularly when students travel to developing countries. Like ‘voluntourists’, the majority of whom hail from developed Western nations [26], medical students usually share demographics in being young, mobile, relatively affluent, educated and with Western visa and other privileges.

Similarly, the critique of the ‘altruistic’ motivations of voluntourism as in fact CV-building and personal, cultural and professional development, whose benefits to host communities are ‘assumed rather than researched’ [25], can also apply to medical electives. These learning experiences are of course also part of the UK medical curriculum and a valued tradition among generations of doctors since the 1970s [4, 27]. Much of the deliberation during the RSM meeting described in this paper reinforced the common belief that as well as an educational experience, many medical students seek a holiday or holiday-like destination for their elective clinical attachment, making tropical and low-income destinations very popular.

This has long been seen as a problematic element of HIC medical electives, which observes this ‘perverse subsidy’, whereby clinicians and others in LMIC settings can devote disproportionate amounts of their time teaching and concerning themselves with the visiting HIC medical students on elective to the possible detriment to their own students whilst not receiving much in return – either personally or institutionally. As mentioned above, MSIH has made longstanding and reasonably successful efforts to deal with such challenges. However the unstructured nature of the typical UK medical elective, which is ‘very much up to you where you go and what you do’, as promised by the British Medical Association’s online resource for student electives (2019), and with no requirement for an established relationship between sending and receiving institutions, undoubtedly favours this imbalance.

Indeed, Dowell and Merrylees [4] identified concerning features among medical electives such as patient vulnerability, ‘learning at the expense of patients’ and ‘exploiting vulnerable health care systems’, when medical students find themselves with ‘exceptional responsibilities’ well beyond their competence. This is in direct breach of one of the central tenets of medical professional bodies such as the GMC’s *Duties of a Doctor* (2019), which implores medical professionals to ‘recognise and work within the limits of your competence’, not to mention undermining the first principle of *do no harm*. From a post-colonial perspective, such educationally exploitative practice is very much in line with Spivak’s critique of the West utilising the Global South as a ‘resource’ from which to draw knowledge that preserves the ‘Western academy and the Western academic at the centre’ [28].

Poor clinical and ethical practices whilst on elective could well compromise the high professional standards students are expected to adopt and maintain before they have even begun life as fully-fledged doctors. Moreover, even if medical students do not bring poor practices home, they may still form a culturally relativist view of following strict protocols such as those in the UK or a similar environment but not so much when working in a different healthcare system with fewer accountability protocols and lower patient agency.

On the positive side however, if planned and executed properly, we see *structured* electives as a mechanism for avoiding voluntourism and the temptations of culturally relativist medical practices. Partnerships developed according to the aforementioned principles in particular can be a very useful tool to utilise medical elective resources appropriately and as a more sustainable health resource for compensating host institutions, particularly in the LMIC context. If handled adroitly, such electives can encourage learning in both directions.

### Proposed future actions

We argue that in HICs such as the UK, medical schools should begin to look seriously at the development of a more structured medical elective, and if this is impossible due to time and resource constraints, many other steps can be taken to improve the quality of pre-departure training and sensitisation. As such, UK Medical Schools Electives Committee published a consensus statement with recommendations for UK undergraduate medical electives [29]. We argue that these recommendations are vital for institutions in the UK and other HICs to consider, but that ultimately they do not go far enough to remedy some of the issues we have identified in this paper.

## Conclusion

In this article we have made an effort to move the debate forward concerning ‘away’ rotations. Our primary goal was to highlight the importance of a structured medical elective as a potential corrective to the many drawbacks of individually organised experiences. While we appreciate that some students may view our prescription as needlessly restrictive, we argue that in terms of equity and ensuring positive arrangements between sending and receiving institutions, these are necessary developments. Likewise, looking more deeply at ethical issues surrounding medical electives as a whole, including voluntourism, should enable us to conceptualise electives differently, and potentially even to influence how they are run. This endeavour therefore requires significant engagement from medical schools in the UK and other high-income countries.

## Data Availability

Not applicable.

## Abbreviations

BMA: British Medical Association
FCO: Foreign and Commonwealth Office
GH: Global Health
GMC: General Medical Council
HIC: High-Income Country
IME: International Medical Elective
LIC: Low-Income Country
LMIC: Low and Middle-Income Country
MSIH: Medical School for International Health
RE: Responsible Elective
RSM: Royal Society of Medicine
